# Cold Adaptation Strategies and the Potential of Psychrophilic Enzymes from the Antarctic Yeast, *Glaciozyma antarctica* PI12

**DOI:** 10.3390/jof7070528

**Published:** 2021-06-30

**Authors:** Nur Athirah Yusof, Noor Haza Fazlin Hashim, Izwan Bharudin

**Affiliations:** 1 Biotechnology Research Institute, Universiti Malaysia Sabah, Jalan UMS, Kota Kinabalu 88400, Sabah, Malaysia; nrathirah.yusof@ums.edu.my; 2 Water Quality Laboratory, National Water Research Institute Malaysia (NAHRIM), Ministry of Environment and Water, Jalan Putra Permai, Seri Kembangan 43300, Selangor, Malaysia; hazafazlin@nahrim.gov.my; 3 Department of Biological Sciences and Biotechnology, Faculty of Science and Technology, Universiti Kebangsaan Malaysia, Bangi 43600, Selangor, Malaysia

**Keywords:** Antarctica, antifreeze protein, cold-active proteins, cold adaptation, membrane fluidity, psychrophilic yeast

## Abstract

Psychrophilic organisms possess several adaptive strategies which allow them to sustain life at low temperatures between −20 to 20 °C. Studies on Antarctic psychrophiles are interesting due to the multiple stressors that exist on the permanently cold continent. These organisms produce, among other peculiarities, cold-active enzymes which not only have tremendous biotechnological potential but are valuable models for fundamental research into protein structure and function. Recent innovations in omics technologies such as genomics, transcriptomics, proteomics and metabolomics have contributed a remarkable perspective of the molecular basis underpinning the mechanisms of cold adaptation. This review critically discusses similar and different strategies of cold adaptation in the obligate psychrophilic yeast, *Glaciozyma antarctica* PI12 at the molecular (genome structure, proteins and enzymes, gene expression) and physiological (antifreeze proteins, membrane fluidity, stress-related proteins) levels. Our extensive studies on *G. antarctica* have revealed significant insights towards the innate capacity of- and the adaptation strategies employed by this psychrophilic yeast for life in the persistent cold. Furthermore, several cold-active enzymes and proteins with biotechnological potential are also discussed.

## 1. Introduction

Over 70% of the Earth’s biosphere are persistently cold environments, which comprises the glaciers, frozen soils, deep ocean and polar sea ice. Antarctica, located at the southernmost part of the Earth, is among the world’s most extreme environments, experiencing strong winds, very low atmospheric humidity, high incidences of solar and especially ultraviolet radiation (UV) and low precipitation and temperatures [1,2,3]. Furthermore, Antarctica is the only continent that is continually blanketed in ice sheets and contains 80% of the earth’s glacier ice. About 15% of the continent is covered by sea ice, which constitutes an important habitat for organisms in the region. These persistently cold and often inhospitable conditions of sea ice have created unique ecosystems, shapes its biological diversity and resulted in one of the most exclusive habitats on Earth [4]. Sea ice is a dynamic composite material composed of liquid brine within a matrix of pure ice crystals [5]. The presence of permeable channels of brine in the semi-solid matrix of frozen seawater allows the growth of sea ice organisms. Ice-associated microorganisms, in particular, play an important role in nutrient (carbon and nitrogen) cycling, recycling and are the beginning of polar food webs, which affects all trophic levels [6]. Although ice-associated microorganisms are crucial in regulating natural ecosystems and ultimately affect climate change, they are rarely the focus of and are not considered in policy development.

**Figure 1:**
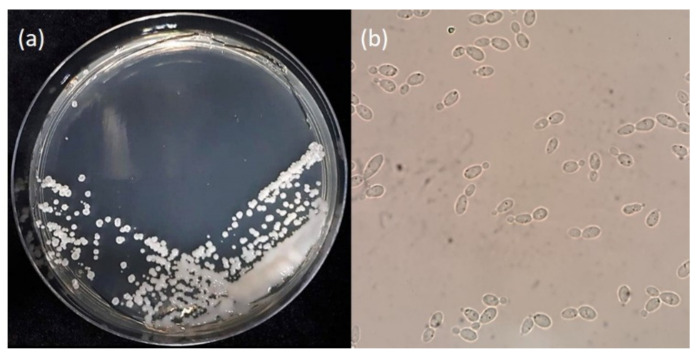
The morphology of *G. antarctica* grown on Yeast Peptone Dextrose (YPD) agar at 12 °C for 10 days. (**a**) The presence of exopolysaccharides on the surface of the yeast cells; (**b**) *G. antarctica* cells observed under a light microscope (40× magnification).

*Glaciozyma antarctica* was isolated from sea ice near the Casey Research Station (66°21′025″ S; 110°37′09″ E), Antarctica [7]. In its native Antarctic environment, this yeast lives in marine waters with temperatures ranging from −2 to 10 °C. However, laboratory experiments have shown that the optimum growth temperature for this yeast is 12 °C, but it can also survive below freezing temperatures (<0 °C) and the highest temperature of 20 °C [7]. The Antarctic yeast was first known as *Leucosporidium antarcticum*, however it has been reclassified to *Glaciozyma antarctica* based on phylogenetic analyses of the D1/D2 region of the large-subunit (LSU) rDNA and its physiological and morphological characteristics, including the inability of this yeast to produce lenticular bodies [8]. *Glaciozyma* was named based on the isolation area—which is from ice and related cold habitats—and currently, only three *Glaciozyma* species has been characterized; *Glaciozyma martini*, *Glaciozyma watsonii* and *G. antarctica* [8]. When grown on an agar medium, *G. antarctica* produces carbohydrate polymers known as exopolysaccharides to protect the cell against freezing temperatures [9] (Figure 1). The *G. antarctica* genome is around 20.03 Mb, with 7857 protein-coding genes [10]. A better understanding of *G. antarctica*’s life in cold habitats has recently been accomplished by the collection of extensive years of studies that gives information at the gene level until a protein is formed. The incorporation of genome data, gene expression and proteome analysis, protein expression and functional and structural analysis has enhanced our knowledge of this yeast significantly. Hence, the study of polar microorganisms will lead to a better understanding of Earth’s biosphere, in hopes of creating an environmentally sustainable future [11].

This review paper will present an overview of various strategies employed by *G. antarctica* in cold adaptation. Its innate adaptive capacity to cope with life in the persistent cold and the associated stresses that accompany such a lifestyle positions it as a potential model organism not just representing psychrophilic yeast populations but also other microbial lifeforms in the Antarctic cryosphere. Furthermore, a number of cold-active enzymes/proteins with high potential in biotechnological industries and the prospect of using this yeast as a biomarker to anticipate the biological fate of Antarctic marine biomes under future climate change scenarios will also be highlighted.

## 2. Molecular Adaptation

### 2.1. Genome Structure

The advancement of sequencing technologies has improved our understanding of psychrophile biology. To date, a number of psychrophilic microbes, including bacteria and fungi, have been sequenced from both polar regions [2,12]. Recently, the whole genome of a few psychrophilic yeasts has been sequenced including *Glaciozyma antarctica* [10], *Mrakia hoshinonis* [13] and *Candida psychrophile* [14].

The *G*. *antarctica* genome size is ~20 Mb, which is comparable to those of the two well-studied basidiomycetes *Cryptococcus neoformans* [15] and *Ustilago maydis* [16]. However, the genome size of this yeast is considerably larger compared to other psychrophilic bacteria such as *Halorubrum lacusprofundi* ACAM34 (3.69 Mb) [17], *Sphingomonas* sp. strain UV9 (4.25 Mb) [18] and *Pseudomonas* sp. MPC6 (7.22 Mb) [19] and the mesophilic yeast *Torulaspora delbrueckii* (9.52 Mb) [20], whereas it is smaller as compared to other psychrophilic fungi such as *Cryomyces antarcticus* (24.32 Mb) [21] and *Rachicladosporium antarcticum* CCFEE 5527 (47.4 Mb) [22]. However, the genome size of this yeast is considered moderate as compared to other psychrophilic yeasts such as *Candida psychrophile* (11.2 Mb) [14], *Dioszegia cryoxerica* (39.5 Mb) and *Mrakia* *psychrophila* (27.8 Mb) [23] (Table 1).

While the general opinion is that overall genomic GC content does not aid in distinguishing between microbial environmental origins and thermal classes, the variation in GC content of the psychrophilic genomes is likely influenced by environmental factors such as temperature and habitat [30]. Interestingly, the overall GC content of this yeast is considerably high, i.e., about 60% [10], which is higher than the other psychrophilic yeasts *M. psychrophila* (53.8%), *D. cryoxerica* (56.1%) [23] and *C. psychrophila* NRRL Y-17665^T^ [14]. However, the recorded GC content of several other psychrophilic bacteria is higher than that observed in the *G. antarctica* genome, such as *Arthrobacter* sp. TB23 (63.32%) [27] and *Sphingomonas* sp. strain UV9 (65.62%) [18] (Table 1). No direct correlation, however, has hitherto been shown to exist between psychrophiles and their genomic GC content.

Gene prediction and annotation of the *G. antarctica* genome has identified 7857 putative protein-coding sequences (CDS), whereas expressed sequence tags (ESTs) generated from various treatments (temperature, media and growth phases) yielded 7369 unique transcripts [10]. The EST data generated showed high similarity (about 67%) with the predicted coding regions from the assembled genome whereas the remaining 37% were categorized as hypothetical proteins. This shows the importance of global gene expression analyses such as ESTs, RNAseq and microarray in validating the gene prediction analyses. Another psychrophilic yeast, *C. psychrophile*, has a slightly smaller number (5827) of predicted protein-coding genes [14]. Genome mining analyses have identified 12 major groups of chaperones which consist of 89 genes encoding for heat-shock proteins or molecular chaperones [31]. These groups of chaperones contribute to the cold-adaptation mechanism via various intracellular processes that encompass molecular function, cellular components and biological processes.

The *G. antarctica* genome also harbors 3 rRNA genes and 79 tRNA genes [10]. Moreover, comparative genomics analyses with other fungal genomes including *C. neoformans* and *Saccharomyces cerevisiae* (mesophilic yeast), *Chaetomium thermophilum* and *Myceliopthora thermophile* (thermophilic fungi) and *Pseudogymnoascus destructans* (psychrophilic fungi) revealed that 31.4% of the genes were exclusive to the *G*. *antarctica* and could possibly be associated with psychrophily [10]. Another important finding from the genome analyses is that about 4% (294) of the annotated open reading frames (ORFs) are smaller than 100 amino acids and are termed as short ORFs (sORFs). Interestingly, 54% (159) of the sORFs are unique in *G. antarctica* [32], thus signifying a possible role in the adaptation of this yeast to the cold environment in Antarctica.

### 2.2. Cold-Adapted and Cold-Active Enzymes

Almost every important structure within living cells, such as the nucleus, cell membrane and cytoplasm, are made of protein building blocks. These building blocks contain enzymes that control biological and biochemical reactions that are vital for the modulation of cellular responses and structures. Hence, proteins are often categorized based on their function as either active proteins or structural proteins. Enzymes are active proteins that function as biological catalysts controlling thousands of chemical reactions inside a living cell. On the other hand, collagen and keratin are the main structural proteins contributing to the structural properties of the cells. Extensive studies on protein function have shown that most cold-adapted enzymes display higher catalytic efficiency (k_cat_/K_M_) compared to their mesophilic counterparts as the temperature is lowered, at the expense of thermal stability [33,34,35,36].

To date, cold adaptation strategies in proteins—and more importantly on enzymes—are linked to increased flexibility in protein architecture either in the area of the active site or in a more distant part of the structure that plays an important role in protein conformational changes [36,37]. Another key factor is amino acid substitutions in key regions of the protein, where frequent substitution by glycines is seen particularly at the loop regions [38,39]. Moreover, cold-adapted proteins are found to have a decrease in the protein structure stabilizing interactions such as hydrogen bonds, salt bridges, ionic and aromatic interactions in either intra- or inter-protein interactions [39]. Nevertheless, there is no general code for the establishment of cold-adapted properties as every protein has its way of enhancing its catalytic activity at low temperatures.

Our studies on *G. antarctica* have shown the existence of similar adaptation strategies and of a few unique strategies that were possibly acquired through selective evolution. One example of these findings—through comparative analysis—is that the cold-adapted chitinase of *G. antarctica* has lesser salt bridges and hydrogen bonds, which increases protein structure flexibility, enabling it to function more efficiently in the cold. Besides that, *G. antarctica* chitinase exhibits a higher exposure of hydrophobic side chains to the solvent and an increase in the charged accessible surface area when compared to its mesophilic counterpart. These findings are linked to the increment of structure flexibility under cold conditions [40]. The increment of structural flexibility in cold adaptation is also supported by other findings such as *G. antarctica* esterase [41], exo-b-1,3-glucanase [42], laminarinase [43], β-mannanase [44], α-amylase [45] and lipase (HSL)-like esterase [46]. Structural elucidation of *G. antarctica* fuculose aldolase using X-ray crystallography data shows that the enzyme retains a highly conserved catalytic histidine triad, an increase in its non-polar residues on the protein surface and a lower number of arginine residues, features that serve for adaptation in the cold [47].

Meanwhile, our study on the structure of molecular chaperones revealed several interesting findings that are potentially unique to *G. antarctica*; for example, Sgt1, which is a homolog to heat-shock protein 20 (HSP20), contains high alanine substitutions at the twist and loop regions. Instead of hindering the stabilization interactions, the alanine substitutions reduced the number of ionic and hydrogen bonds. Besides, these changes also decreased the number of aromatic interactions in the structure, leading to a concomitant increase in the molecular flexibility of the protein and facilitating conformational modifications associated with protein binding and decreasing the strength of inter-protein associations [48]. Another study focusing on a mega-sized *G. antarctica* protein chaperone, the TCP1-1 ring complex (TRiC), showed a complex adaptation strategy that featured the substitution of amino acids in the conserved regions. Some of the conserved amino acids were substituted with a member of the same amino acid group, that led to an increase in the distance between residues that contribute to protein stability via ionic, hydrophobic and aromatic interactions [49]. Another intriguing finding is the increased number of aromatic interactions in *G. antarctica* cold shock protein and the clustering of charged amino acids on the protein surface. It is postulated that with the presence of a longer loop between nucleic acid binding domains in the *G. antarctica* cold shock protein compared to its mesophilic counterparts, the flexibility of the protein structure is efficiently modulated while retaining its structural stability [50]. These findings show that, like other psychrophiles, *G. antarctica* proteins have evolved a range of structural features that leads to increase flexibility to account for higher catalytic efficiency at the expanse of structural and thermal stability.

### 2.3. Gene Expression

Gene expression analysis is important to determine the physiological and environmental effect on intracellular changes [9]. Several techniques have been adopted in quantifying these changes, such as microarray, ESTs and RNAseq. Ten different EST libraries were generated from different conditions such as growth temperature, media and growth stages [10]. Three different libraries were generated from three different exposure temperatures (−12, 0 and 15 °C); to mimic their natural temperature environments during different seasons [4].

A study on the adaptation of *G. antarctica* in different growth mediums was also carried out utilizing two growth media: a complex medium (yeast peptone dextrose, YPD) and a minimum medium (yeast nitrogen base, YNB) [51]. In total, 1492 and 1928 unique transcripts were generated from complex and minimum medium, respectively. Interestingly, several genes that are important for free amino acid uptake were highly expressed in the complex medium treatment, such as the gene encoding for general amino-acid permease (*GAP1*) and proline-specific permease (*PUT4*). In the minimal medium treatment, the genes which showed higher expression were essential for biosynthesis and recycling of amino acids such as GMP synthase (*GUA1*) and cell wall biogenesis protein phosphatase (GUL*1*). These results show that *G. antarctica* utilizes all amino acids from the environment by increasing the production of permease, which is important in collecting free amino acids. However, during nutrient limitation, the yeast will synthesize the amino acids needed or recycle peptides or proteins to scavenge or gain access to required amino acids [51].

Furthermore, with the advance of sequencing technologies, a transcriptomic study was generated using the RNAseq platform to eliminate any bias possibly introduced during the process of EST sequencing. Twelve RNA-seq samples with 325,038,726 reads were generated. From these reads, 50.11% of the sequences were represented in the annotated genome whereas 11.08% was not mapped to the whole genome sequence [52]. Comparative transcriptomic analyses have identified the overexpression of common genes under three tested conditions (‒12, 0 and 12 °C) for instance HSP70 and HSP90, ribosomal proteins, eukaryotic translation initiation and cyclophilin. Interestingly, several regulatory genes and chaperones that were important for optimal growth and survival at freezing temperatures such as RNA helicase, heat shock proteins, cold shock domains and peptidylprolyl isomerase (PPIase) were constitutively expressed [52]. Interestingly, four cold-shock domain-containing proteins and nineteen genes encoding PPIase were identified. Both groups of proteins are involved in activating transcription factors and protein folding by binding to a specific nucleic acid [53].

Another group of genes that are important in cold adaptation were also found in the transcriptome studies, such as genes encoding for antifreeze proteins (AFPs). The AFPs play an important role to prevent the formation of intra- and extra-cellular ice-crystallization, thus enabling the cell to withstand fluctuating freezing temperatures, and consequently, cell death [54,55]. *G. antarctica* has nine genes encoding for AFPs and the gene expression analyses revealed that each *AFP* has diverse expression levels, depending on various factors including organism, temperature and environment [4,10,52,54]. Interestingly, only two AFP-encoding genes were identified in the EST analyses with one transcript encoding AFP7 (*GaAFP7*), which was detected from the −12 °C library, and one transcript encoding AFP9 (*GaAFP9*), which was identified from the 0 °C library [4]. The data was supported with qPCR analysis, which showed similar expression levels.

Besides, environmental stressors such as heavy metal pollutants and freezing can activate the production of reactive oxygen species (ROS), causing intracellular DNA damage [7,23,56,57]. To overcome this, *G. antarctica* produces several superoxide dismutases, where mainly manganese superoxide dismutase (*MnSOD*) and peroxiredoxin (*prx*) encoding genes were constitutively expressed [52]. Similar results were observed in another psychrophilic yeast, *M. psychrophilia*, where the superoxide dismutase genes were not upregulated [23]. To overcome environmental stress, *G. antarctica* expresses genes encoding for glutathione S-transferase (*GST*), which are important to cleanse ROS formed during metabolism [58,59]. GST also plays an important part in cell protection from oxidative burst by catalyzing various substrates and quenching reactive molecules.

Furthermore, the EST and RNAseq analyses have identified another 319 and 82 respective unique transcripts with unknown functions and currently unique to *G. antarctica* [4,52]. Further characterization analyses have determined that 72% of the unique transcripts from the RNAseq do not have any functional domains [52]. Interestingly, two of the unique transcripts from the EST analysis were up-regulated in the −12 °C library as compared to higher temperatures of 0 and 15 °C [4]. Thus, these findings might support the importance of global gene expression in enhancing knowledge of the molecular mechanisms underlying *G. antarctica* cold adaptation in their natural environment.

## 3. Physiological Adaptation

### 3.1. Antifreeze Proteins

One of the key elements that enable psychrophiles to thrive in cold temperatures is the production of cold-tolerance proteins called antifreeze proteins (AFPs). These proteins have affinity towards ice and are able to reduce the freezing point of a solution in a non-colligative manner, without altering the melting point of the solution temperature [60]. This process, termed thermal hysteresis (TH), also alters the ice shape formation depending on the binding site of the proteins to the ice [61]. Apart from that, AFPs function by inhibiting the nucleation and growth of ice crystals that can cause cell damage, a process called ice recrystallization inhibition (IRI) [62]. These two features are the main characteristics of AFPs.

Since its first discovery from arctic fish, AFPs have been reported from different types of organisms, varying from plants, fishes, insects, diatoms and microbes. Albeit, AFPs play different roles in different organisms and can be classified based on whether they promote avoidance to freezing, i.e., preventing freezing altogether; or promoting tolerance to freezing, i.e., preventing the damage of freezing, but not freezing altogether. Generally, AFPs adhere to ice crystals, inhibit their growth and help in restructuring ice formation/recrystallization, and thus preventing the organism from freezing. Insect AFPs for instance, are known to have high TH activity even up to 7 °C [63]. It is critical for the organism to have high TH activity for it to maintain inner cell fluidity and from freezing in subzero temperatures (freeze avoidance). In contrast, microbial AFPs are known to have high RI activity compared to TH activity. This allows microbes to tolerate the freezing temperature; where most of the proteins are produced extracellularly to maintain the water channels in sea ice to enable nutrient uptake, proliferation and eventually survival in the cold habitat [60].

**Figure 2:**
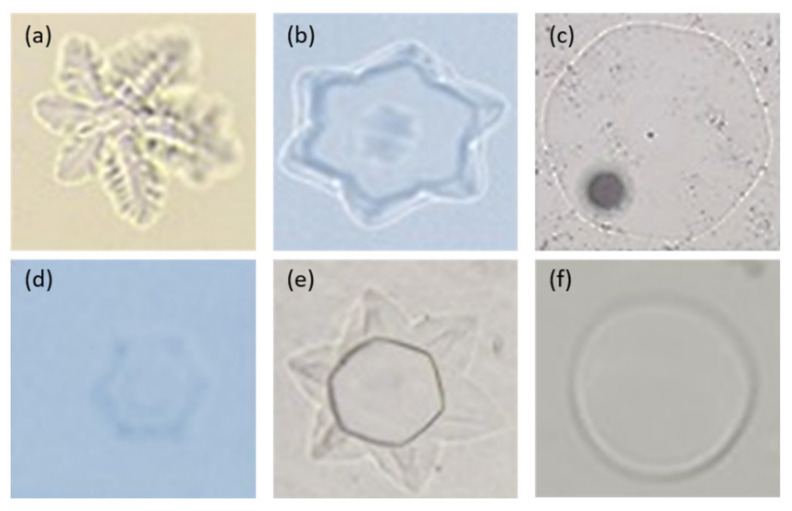
The formation of ice crystals exhibited by recombinant GaAFPs. Ice crystals in samples containing a mixture of recombinant GaAFP: (**a**) all recombinant GaAFPs; (**b**) GaAFPs with high TH activity (more than 0.05 °C); (**c**) GaAFPs with low TH activity (less than 0.05 °C); (**d**) GaAFPs with moderate TH activity (0.05 °C); (**e**) GaAFPs with high and low TH activity; (**f**) control treatment containing proteinase K.

*G. antarctica* is reportedly able to secrete AFPs when cultured at cold temperatures [55]. Secreted proteins in the culture media exhibited low TH activity at 0.1 °C and high RI activity. The genome sequence of the yeast revealed nine genes encoding for antifreeze proteins (GaAFP) and they exhibited different effects on the shape of ice crystal formed [8]. Moreover, the different mixtures of individual recombinant AFPs also exhibited a variety of ice crystal shapes (Figure 2). The activity for each GaAFP produced recombinantly showed low TH activity (0.05–0.08 °C) and high IRI activity [10]. Although the sequence length varies, it shares one common domain characteristic, which is the Domain of Unknown Function (DUF3494). This domain with an unknown function is also detected in other microbial AFPs [64,65]. Domain DUF3494 has been found in microbial AFPs and numerous proteins containing this domain have been found to bind ice crystals, leading members of this protein family to be recognized as the ice binding-like family; although there is little conservation in the amino acid sequence [66,67]. Most DUF3494 AFPs contain N-terminal signal peptides; indicating that the expressed protein is localized extracellularly to minimize the effect of ice crystals towards the cell [68].

**Figure 3:**
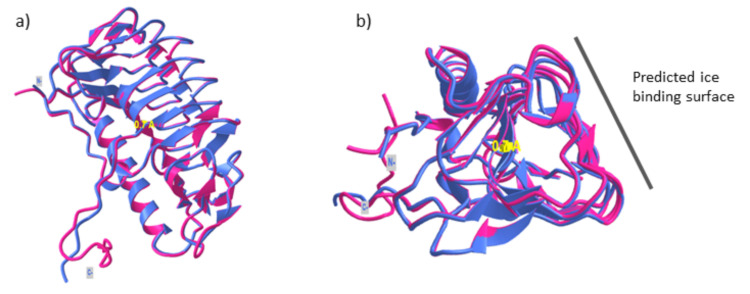
(**a**) Superimposition of GaAFP4 predicted structure (magenta) and LeIBP (PDB id: 3UYU) (purple) with RMSD value 0.7 Å. The structure showed a typical β-helical fold as reported in other fungal AFPs. (**b**) Predicted ice-binding surface of GaAFP4.

AFPs are a structurally diverse group of proteins, although they possess the same function, i.e., to prevent ice crystals growth. Currently, there are ~11 different folds of three-dimensional protein structure that have been reported. Microbial AFPs show a typical triangular β-helical fold with a stretch of α-helical structure alongside the triangular fold; this is markedly different compared to AFPs produced by other organisms. This structurally diverse group of proteins has been suggested to arise from the evolutionary process such as horizontal gene transfers that have enabled these organisms to survive in the harsh environment [69]. The structure of one of the GaAFPs, AFP4, showed that AFP4 folds into typical β-helices with three distinct planes [70]. Superimposition with the 3D structure of LeIBP (PDB id: 3UYU) showed high similarity between the predicted structure of GaAFP4 with RMSD value 0.45 Å indicating high similarity between both structures (Figure 3). The structural comparison showed that the ice-binding surface of GaAFP4 at the β-face and the protein-surface interaction is driven by hydrophobic interactions [71]. The ice-binding surface is predicted to be on the flat β-sheet face based on the hydrophobicity value. There are no distinct repetitive patterns/motifs that function as ice-binding residues; compared to what has been reported in other microbial AFPs. However, a 4.7 Å uniform distance between each of the β-sheets on the predicted surface binding complements the distance between the oxygen atom of each water molecule.

### 3.2. Membrane Fluidity

The cell membrane plays a vital role in nutrient uptake, signaling, energy transduction and acts as the first barrier from any environmental stressors [72]. The cell membrane is in a rigid form in freezing temperatures, thus inactivating the function of certain transmembrane proteins including carrier and transporter proteins [73].

Several known genes responsible for cold adaptation such as the delta-9 and delta-12 fatty acid desaturases (FAD) were upregulated at the low temperature of −12 °C. The addition of the first and second double bond to the fatty acid (FA) chain is dependent on the presence of both FADs [4]. Not only that, the FA profiles also showed that the majority of the FAs is in the form of unsaturated FA (UFA). From that, about half of it contains a single double bond, in the form of oleic acid (C18:1). Interestingly, the polyunsaturated FA (PUFA) (C18:2 and C18:3) content increased by 1–2% to fuel the membrane fluidity particularly at the freezing temperature of −12 °C [4,10]. Similar findings were also observed in other psychrophilic microorganisms such as the bacterium *Shewanella* sp. *GA-22* [74] and archaeon *Methanococcoides burtonii* [75].

Further analysis on the FA content revealed that *G. antarctica* also produced *trans*-form FAs such as elaidic acid (C18:1T) and linolelaidic acid (C18:2T), even though the majority of FAs produced were in the *cis*-form such as oleic acid (C18:1C) and linoleic acid (C18:2C) [4]. The isomerization of *cis* to *trans* UFAs is influential in changing the fluidity of the membrane, thus enabling the psychrophilic yeast to adapt to the stress of environmental freezing. Furthermore, previous study also showed that deletion of RKD12 (PUFA gene) in yeast *Rhodosporidium kratochvilovae* YM25235 hinder the fluidity of its membrane and decrease the amount of PUFA especially at low temperature [76]. Hence, this indicates the important roles of FADs in preserving the integrity of the cell membrane as a rational modification in response to extremely low temperatures.

### 3.3. Stress-Related Proteins

Extreme cold environments are associated with various environmental stressors including extreme temperature downshift, low nutrient availability, radiation, excessive UV and high osmotic pressure [77]. Psychrophiles have to develop internal protection such as producing stress-related proteins that sense the internal and external changes and that respond to these stressors. Previous studies have shown that psychrophiles produce HSPs, cold shock proteins (CSPs), cold-active enzymes and molecular chaperones to encumber these stressors [36,57,78,79,80].

Studies on the *G. antarctica* genome, transcriptome, and proteome have shown that this psychrophilic yeast produces molecular chaperones when exposed to thermal stress. Our genome analysis shows the presence of RNA chaperones, HSPs and peptidyl-prolyl isomerases (PPIase). Interestingly, comparison at the genome level with non-psychrophilic yeasts such as *S. cerevisiae*, *C. neoformans*, and *C. thermophilum* shows the presence of proteins related to the cold stress response that were not found in the compared genomes. These proteins include four genes encoding CSPs, one PPIase and six HSPs. Since these protein-coding genes are found in the psychrophile, *P. destructans*, this shows that these protein-coding genes are therefore possibly associated with cold adaptation strategies employed by psychrophilic fungi [10]. Besides, genome mining also shows the presence of 89 possible molecular chaperones consisting of TRiC chaperonin, HSF proteins, HSP70, HSP40, HSP20, HSP90, CSPs, AAA proteins, CS-domain proteins, ubiquitins and tetratricopeptide repeat (TPR) domain proteins [31]. Moreover, proteome analysis shows the presence of a few chaperones related to HSPs and PPIase when *G. antarctica* cells were exposed to cold stress. In parallel to these findings, gene expression analysis supports the importance of molecular chaperones particularly for heat and cold adaptation strategies. Boo et al. (2013) reported significant changes in HSP70, HSP90 and HSP100 when *G. antarctica* cells were exposed to 0 and 22 °C [7]. These findings show the importance of HSPs for protection against environmental stress and survival in the cold.

Another intriguing finding is the presence of expansin-like proteins in *G. antarctica*, the first of its kind from a psychrophilic microorganism. Expansin proteins play a vital role in loosening and softening the cell wall during cell expansion by disrupting the non-covalent bonds between matrix glucans and cellulose microfibrils, causing extension of the cell wall [81]. *G. antarctica* secretes this protein and it may be targeted towards polymeric substrates in sea ice to accelerate the substrate loosening processes. The protein helps in efficient substrate hydrolysis and acts as one of its adaptation strategies in the sea ice environment [82]. Furthermore, *G. antarctica* has also been reported to produce cold-adapted enzymes that have increased structural flexibility, lower thermostability and higher specific activity at low temperatures compared to their mesophilic counterparts.

A study on the *G. antarctica* esterase showed that the cold-active enzyme performs optimally at 10 °C and retains more than 50% of its function in the temperature range of 0–30 °C and is stable in the pH range of pH 5–10 [41]. Concomitantly, *G. antarctica* chitinase performs optimally at 15 °C and was able to retain its activity between 5–25 °C at pH 3–4.5 [83]. Interestingly, we found several intriguing and unusual findings where some *G. antarctica* enzymes deviate from common psychrophilic properties. As an example, the *G. antarctica* lipase-like esterase was found to work optimally at 50–60 °C [46] and cold-active type II 3-dehydroquinate dehydratase displayed maximal activity at 40 °C [84]. These show that at low temperatures, *G. antarctica* adopts its adaptive machinery by producing cold-active proteins such as chaperones and enzymes as part of its strategy of adaptation to low temperatures. However, the molecular basis of the activity-stability-flexibility relationship in psychrophilic proteins, although extensively explained in other research, is still enigmatic and does not apply to all proteins in psychrophiles. Similar to our findings, other studies have found that some cold-active proteins are yet heat-tolerant [85,86,87]. The weakening of protein intra- and inter-molecular forces, which are linked to high flexibility and instability in proteins, in order for these proteins to function efficiently in low-temperature environments may result from random genetic drift due to lack of evolutionary pressure for high enzyme thermostability in the cold. Our findings support the theory of evolutionary positive selection, which explains the balance of cold-active proteins in terms of the activity-flexibility-stability relationship [88].

## 4. Potential Biotech Application

Adaptation strategies exhibited by *G. antarctica* have not only aided towards understanding their survival mechanisms at harsh temperatures, but also represents a promising source for potential biocatalysts. Given their proclivity for high activity at low temperatures and thermolability, cold-active enzymes/proteins are superior candidates for numerous industrial applications. Apart from its structural characteristics, cold-active enzymes/proteins trump their mesophilic counterparts as it minimizes energy usage and reduces the need for high temperature to activate the enzymes, thus leading to more economical and sustainable processes [89].

Usage of antifreeze proteins as biological antifreeze agents has piqued high interest, especially in the medical and food industries. Due to their unique affinity towards ice, AFPs can be applied as cryoprotectants in the cryopreservation process. Kim et al. (2017) have extensively reviewed the potential use of antifreeze protein as a cryoprotectant compared to the conventional process using liquid nitrogen. The ability of AFPs to decrease the temperature of the solution and inhibit ice grains gives advantages in alleviating cell injury during the thawing process. In food industries, the application of Type III AFPs has been patented by Unilever in ice cream production to improve the texture by preventing ice grain formation (US Patent US6914043B1). Additionally, application of a fungal AFP from the *Leucosporidium* ice-binding protein (LeIBP) in Korean beef preservation showed delays in lipid peroxidation, hence prolonging the shelf life of the meat. Apart from that, LeIBP helps to maintain the meat quality by preventing microbial growth and improves the activities of the antioxidative enzymes during storage [90]. Recombinant production of GaAFP1 in *Pichia pastoris* yielded ~40 mg/L protein with high TH activity compared to recombinant GaAFP1 produced in *Escherichia coli* system may lead to potential mass production of this enzyme for industrial applications [91]. The addition of GaAFP1 into cellulose enzymes allowed the cellulases to retain their activity after several cycles of freeze-thawing, showing further promising potential in biotechnological applications.

Another class of enzymes that is exploited in the biotechnological sector is hydrolases [92,93]. Enzymes such as lipase, esterase, glycosidase, protease and chitinase have huge potential to be used in a myriad of processes in the food, detergent, textiles and waste industries. Esterase enzymes play a major role in the degradation of natural materials and industrial pollutants, cereal wastes, plastics, and other toxic chemicals [89]. Hashim et al. (2018) reported a new cold-active esterase-like protein with putative dienelactone hydrolase (GaDlh) function with an optimal temperature of 10 °C and an optimum pH of 8.0 using short-chain soluble esters as substrates. However, due to the lack of a specific substrate commercially, the specific function of GaDlh remains unknown. Proteases are one of the key enzymes widely used in most industries, particularly in food industries. Cold active proteases have immense potential due to their variety of industrial applications such as cheese ripening, meat tenderization and fish descaling, where the process needs to be carried out at low temperatures [92]. *G. antarctica* showed its ability to produce proteases extracellularly by hydrolyzing casein substrate in the culture media [94]. The recombinant protease produced in *P. pastoris* showed the highest activity at 20 °C and decreased tremendously after 35 °C due to the denaturing state of the enzyme [94]. Another subtilisin-like protease in *G. antarctica* (GaSUB) which is categorized under family S8 protease showed a low number of arginine residues, a high percentage of polar residues, less hydrophobic interactions and low glycine content [95]. Glucosidases also have a high potential to be used in food and beverage production, biological agent control, detergents, pharmaceuticals and paper manufacturing [89].

Genome mining of *G. antarctica* showed ~97 putative glucosidase-encoding genes that are primarily comprised of extracellular enzymes, including endoglucanases, xylanases and chitinases [96]. Structural predictions of the putative proteins β-glucanase (laminarase), α-amylase, glucoamylase and mannanase showed typical cold-active enzymes characteristics that can be observed in the predicted protein structures [43,44,45,97]. Biochemical characterization of these enzymes may provide more information on their function at low temperature. Recombinant *G. antarctica* chitinase (ChI II) exhibited high activity ranging from 5–25 °C and is able to maintain at least 50% of its activity at 25 °C [83].

The production of recombinant enzymes is one of the bottleneck issues for mass production in industries. The enzymes are either often produced as inclusion bodies (in prokaryotic expression system) or are low in yield. Expression at low temperatures gives advantages particularly by reducing the formation of inclusion bodies and native protease activity [98]. Apart from that, utilization of native plasmids (origin of replication) from psychrophiles might help to produce highly soluble and active recombinant proteins [99]. Usage of cold shock protein A promoter (CspA) in an expression vector aided in high soluble recombinant psychrophilic enzymes when being expressed at low temperature [100,101]. Chaperones are ubiquitous proteins that usually help in protein folding to their native conformations. Co-expression with cold-active chaperones, Cpn60 and Cpn10 improved protein production by enhancing its specific activity up to 180-fold compared to proteins expressed at 37 °C [102]. The chaperones later were commercialized as the Arctic Express System by Agilent Technologies. As reviewed in the previous section, a total of 89 putative genes in the *G. antarctica* genome were predicted to encode for molecular chaperones (Yusof et al., 2015). Heterologous expression of GaSGT1, a homologue of HSP20 protein has been reported to be able to protect the cells from heat and cold shock temperature exposure (thermotolerance) where the activity was retained at up to 60% after the exposures (Yusof et al., 2016). These may be potential candidates for the bioprospecting of *G. antarctica* proteins in the industrial enzymes market.

The *G. antarctica* expansin protein which is able to disrupt cellulose complexes may bring great potential in the biofuel production industry. Current practice for biomass treatment using acid and high treatment requires high energy and may be substituted by pretreating the biomass with cold-active expansin-like protein. The functional activity of recombinant GaEXLX1, an expansin-like protein identified from *G. antarctica* was successfully characterized [82]. The protein, which shares 36% identity with *Clavibacter michiganensis* expansin-like protein, is able to disrupt/modify cellulosic surfaces when co-incubated for 24 h. Through binding assay experiments, GaEXLX1 was reportedly able to bind to polymeric substrates tested such as crab chitin and lichenan. Further exploitation of this protein may unlock further insights into its potential application in biofuel industries.

## 5. Future Research

Although advances in the understanding of the mechanisms underlying cold adaptation have emerged from the fulfilment of genome, transcriptome and proteome projects undertaken on *G. antarctica*, there still remains much more to be uncovered. One of the fundamental issues in solving the puzzle of the psychrophilic lifestyle is to determine the function of genes in the genome. Transcriptome and proteome data are useful in identifying possible genes and proteins involved in cold adaptation and stress response. However, the molecular toolbox for functional studies (e.g., gene deletion) in *G. antarctica* has yet to be developed, thus making it harder for such research to come to fruition. The development of genome editing techniques such as the CRISPR/Cas9 system, conventional homologous recombination or even transformation methods is the next logical step to advance knowledge on psychrophiles broadly, and *G. antarctica* specifically. Developing such techniques will be essential for further functional genomics studies of *G. antarctica*.

Although the emerging picture suggests that cold-active enzymes share similar characteristics such as improved flexibility and thermal compensation, each psychrophilic enzyme adopts its own adaptive strategies to perform at low temperatures, suggesting that the adaptative strategies found in psychrophilic proteins are not rigid but instead exists as a continuum. Thus, there is significant impetus to delve deeper into psychrophilic enzymes and their stability–flexibility–activity relationship towards better understanding biocatalysis at low temperatures. The current body of work in terms of protein/enzyme characterization, although significant, is still not enough to uncover greater detail, thus additional research is needed, especially towards generating more 3D crystal structures, site-directed and random mutagenesis experiments, as well as biophysical studies. The *G. antarctica* genome is a treasure trove of unexplored proteins and enzymes that remains to be investigated and constitutes not just an important focus for extremophile biology but also holds tremendous potential for biotechnological applications.

## Figures and Tables

**Table 1:** Summary of the *G*. *antarctica* genome in comparison to the genomes of other psychrophilic bacteria, yeast and fungi.

Microorganism	Genome Size (Mb)	G+C Content (%)	Scaffolds (sc)	Genes	tRNAs	rRNAs	Accession Number	Reference
**Bacteria**								
* Halorubrum lacusprofundi *	3.69	64.00	3	3665	NA	NA	PRJNA343348	[17]
* Psychrobacter * sp. strain G	3.07	42.44	NA	2614	48	12	CP006265	[24]
*Planococcus antarcticus* DSM 14505	3.78	42.09	NA	3840	NA	NA	AJYB00000000	[25]
* Planococcus * sp. PAMC21323	3.19	39.30	2	3171	60	24	CP009129	[26]
* Sphingomonas * sp. strain UV9	4.25	65.62	62	3879	50	3	SCIN00000000	[18]
*Arthrobacter* sp. TB23	3.54	63.32	104	3298	46	6	ASZW01000000 AUPJ01000000	[27]
* Pseudomonas * sp. MPC6	7.22	59.96	NA	6330	69	22	CP034783	[19]
**Yeast**								
*Glaciozyma antarctica* PI12	20.0	60.00	21	7857	79	3	PRJNA202387	[10]
*Candida psychrophila* NRRL Y-17665^T^	11.2	36.74	193	5827	192	NA	FYBW01000000	[14]
* Exophiala mesophila * strain CCFEE 6314	30.43	50.00	207	103,55	NA	NA	NAJM00000000	[28]
*Mrakia psychrophila*	27.8	53.80	1976	5994	NA	NA	PRJNA304674	[23]
**Fungi**								
*Antarctomyces pellizariae* UFMGCB 12416	24.21	49.90	395	8748	NA	NA	WCAA01000000	[29]
*Cryomyces antarcticus*	24.32	53.84	12,491	10,731	NA	NA	AYQD01000000	[21]
*Rachicladosporium antarcticum* CCFEE 5527	47.4	NA	267	18,781	NA	NA	NAJO0100000 NAEU01000000	[22]

NA—not available.

## Data Availability

Not applicable.
